# Supplementary material for: Fossils from Mille-Logya, Afar, Ethiopia, elucidate the link between Pliocene environmental changes and Homo origins
Source: Nat Commun. 2020 May 19;11:2480. doi: 10.1038/s41467-020-16060-8 (PMC7237685; doi:10.1038/s41467-020-16060-8)
Supplement: Supplementary file 1 — Supplementary Information [file 41467_2020_16060_MOESM1_ESM.pdf]

Supplementary Information

Fossils from Mille-Logya, Afar, Ethiopia, elucidate the link between Pliocene environmental changes and *Homo* origins

Alemseged et al.

Supplementary Note 1

Basalt Geochemistry

The basalt sequence in MLP site comprises Gafura Basalt I (GFB-I), Gafura Basalt II (GFB-II), Dame Basalt (DMB), Urai Teli-Garsele Dora Basalt (UGB), Goyana Basalt I (GYB-I) and Goyana Basalt II (GYB-II). These are chronostratigraphic groups that also show rather distinct elemental chemistry as can be seen in the Nb/Y versus Ti/Y and La/Nb versus Nb plots (Supplementary Figure 1,2) and Supplementary Table 1. Supplementary Figures 1 and 2 and Supplementary Table 1 illustrate between group geochemical distinctions indicative of group-specific petrogenetic histories. The oldest (GFB-I & GFB-II) and the youngest (GYB-II) groups contain relatively the highest MgO, and compatible trace elements (e.g. Ni and Cr) and the lowest TiO_2, P_2O_3, and incompatible trace elements (e.g. Nb, Zr and Sr), excepting the DMB, whereas it is vice versa for the second youngest group, GYB-I, and also for the UGB (Supplementary Table 1). Like the GFBs, the DMB samples have lowest TiO_2, Zr and Nb and highest MgO but in contrast they have lower Cr and Ni contents. These features suggest lack of a simple suite-wide differentiation trends and emphasize distinctions related to group-specific petrogenetic histories.

The occurrence of DMB at MLP is likely a northward continuation of the extensive Guda Basalt (GDB) in the Woranso-Mille area; and this is supported by the similarity in the elemental composition and trace element ratios between the two groups (see Supplementary Figures 1,2, Supplementary Table 1).

In the Nb/Y versus Ti/Y plot all the MLP basalts fall within the field for Oligocene to Miocene High-Ti dikes and flood basalts from the northwestern Ethiopian plateau. In addition, the following features are noted: (1) the GFBs and DMB fall within the overlap field between the NW Ethiopian Plateau Oligocene-Miocene High-Ti dikes and flood basalts and the Woranso-Mille basalts, (2) there is close similarity between DMB and GDB-Wormil in that both plot almost in the same position on the field, and (3) the GYB-I in particular fall within the HT2 field of Pik et al (1998) that said to exhibit extreme OIB-like compositions.

Tephra Geochemistry

Glass analyses from the vitric tephras indicate that the majority of sampled tephras have relatively homogeneous calc-alkaline, rhyolitic glasse compositions (Supplementary Table 2). Tephra chemical analyses from the Mile-Logya harea have identified correlations across several sections in disconnected areas of the study area (i.e., the Goyana Tuff), and identified potential external correlates for several of the tephras (Goyana, Urai Teli). The Goyana Tuff is a thick (~1.5 m), widespread pumiceous, aphanitic airfall lapilli tuff with woody pumice fragments; it is identified in several sections of isolated fault blocks. The Urai Teli Tuff is the only tephra analyzed with both preserved glass that can be used for chemical correlation, and crystals that

were used for radiometric dating (sample MLP14/URT-1 is dated at 2.443 Ma; see below). This tephra occurs as a thin, reworked deposits exposed in small outcrops in the Uraitele region.

$^{40}Ar/^{39}Ar$ dating

All but four grains carried to completion in the incremental heating experiments yielded apparent-age plateaus (Figure 1). The 'age plateau' identification algorithm used here delineates the set of contiguous steps encompassing the greatest percent of ^{39}Ar release that exhibit an acceptable MSWD ('mean square of weighted deviates,' with a threshold probability >95% that the observed scatter is caused by analytical error alone and that geological scatter is not demonstrated). A plateau must comprise at least 50% of the total ^{39}Ar release and consist of at least three consecutive steps. The age and uncertainty assigned to the plateau are weighted means (weighted by the inverse variance of each step) and modified standard error (standard error expanded by root MSWD if MSWD >1).

The apparent-age plateaus are plotted as age-probability density functions in Figure 2. Whereas all tuffs exhibit a dominant age population mode, old outliers attributed to xenocrystic contaminants are evident in MLP14/GOY-2 and possible MLP14/HM-2. We use a two-step approach to mitigate outliers, applied universally to all samples. First, distinctly older xenocrysts are excluded from the age population using a procedure that evaluates age gaps in the ordered age distribution[1]. Secondly, outliers are identified based on deviations from the population median ('normalized median absolute deviation,' or 'nMAD' = 2). Following sequential application this procedure, a single mode can be identified in each unit in the younger portion of the age distribution (fig. 2). This mode is interpreted as a representation of the primary eruptive age. We then take the argon systematics treatment further, by applying isotope correlation analysis using the 'inverse isochron,' i.e., $^{36}Ar/^{40}Ar$ *vs* $^{36}Ar/^{40}Ar$ (Figure 3). All plateau steps belonging to primary modes identified above are combined on a sample-by-sample basis in this analysis, for improved statistical power (Table 2). The isochron ages are taken as the best representation of the eruptive age of these tuffs, though it should be noted that there is no statistical difference between the plateau means and the isochron ages at the 95% confidence level.

Magnetostratigraphy

All four samples of the Gafura Basalts that were measured gave high quality results (Supplementary Table 5; Supplementary Figure 6A). GFB 14-1 and GFB 14-2 show normal ChRM directions while GFB 15-1 and GFB 16-1 show clear reversed ChRM directions. GFB 14-1 and 14-2 samples show no indication of normal field overprint and have a ChRM range from 150 to 400 °C. Both GFB 15-1 and GFB 16-1 show a clear normal field overprint up to 250 °C (Supplementary Figure 6A). From the Seraitu Cliff (SC) section, 97 samples were collected in the field of these 82 have been demagnetized. A total of 16 samples were taken from the Seraitu Lake (SL) section of which 15 have been demagnetized. The AF demagnetized samples show a normal field overprint to 5mT and a well-defined ChRM from 15-20 mT to 40-60 mT, indicating a low contribution of hematite to the signal. TH demagnetized samples of the SC section show normal overprint directions up to 150-250 °C with ChRM directions defined from 200-250 °C to 500-600 °C with an occasional low of 400 °C. The 400 to 550 °C seems to indicate titanomagnetites are carrying the remanence (Supplementary Figure 6,C,D). The presence of magnetites is confirmed by the IRM plots which show a saturation above 600 mT (Supplementary Figure 7).

No difference between measurements of the two laboratories was noted. For the most part, the ChRM was determined by a steady decay towards the origin. Unforced line fits with

maximum angular deviation (MAD) < 15° were given quality 1 label. Forced line fits with MAD < 20° and unforced line fits between 15° and 20° were given quality 2, while all samples with MAD > 20° have been rejected. For this study samples showing a normal overprint partially overlapping with the reversed ChRM indicating demagnetization paths along a great circle on stereographic projections have also been neglected (N=1). Both quality 1 and 2 samples are included in the interpretation.

Magnetostratigraphic results from the Gafura Basalts clearly indicate that a reversal took place between the two lowermost flow units exposed in the area. Given the position of this reversal below the Iki-elu Diatomite and Hinti Mageta Tuff-2 (MLP14/HM-2; 2.914 Ma) and the lack of a lower bounding age, combined with evidence for disconformable sedimentation and presently incomplete sampling, we can only interpret this reversal conservatively as any reversal of this polarity prior to the Gauss/Matuyama. Most conservatively, this would suggest the lower boundary of the Kaena Chron (3.12 Ma), but could represent prior reversals such as the lower boundary of the Mammoth Chron (3.33 Ma), the Gauss/Cochiti boundary (4.17 Ma), etc (Gradstein et al., 2012)[2]. Further magnetostratigraphic constraints on the sediments and basalts overlying the Gafura Basalts, and/or radiometric dating of the Gafura Basalts may help to narrow the constraints on this part of the section.

The composite section at Seraitu (sections JGW14-10 and JGW14-8) may show some overlap of the cross-bedded sand that is used to connect these two sections. All samples below 7 meters in the lower section (SL) are of normal polarity indicating a polarity reversal between 7 and 5.9 meters, with reversed polarity through 7 meters (Supplementary Figure 7). The upper section (SC) shows reversed polarity throughout, with the exception of one normal paleomagnetic direction (Supplementary Figure 8). This sample may either have been remagnetized or may represent an excursion but as we have only a single sample it has been ignored. We interpret the reversal in the composite Seraitu sections described above as the Gauss/Matuyama reversal. This is based on its position between $^{40}Ar/^{39}Ar$-dates of 2.914 and 2.576 Ma. The age of the Gauss/Matuyama is still being debated. According to the geological timescale of 2012 an age of 2.581 Ma has been assigned[2]. However, several studies seem to indicate that this age should be slightly older (2.606/2.608 Ma;) [3,4].

Supplementary Figures

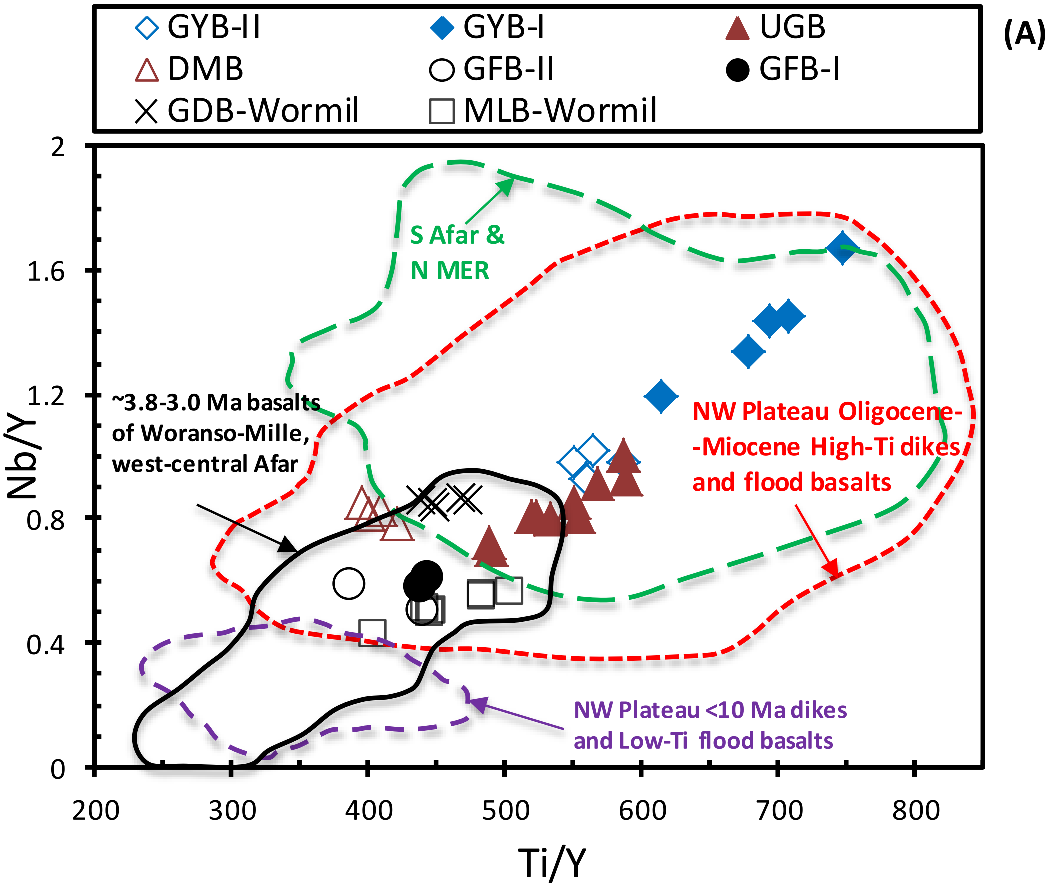

Supplementary Figure 1. Nb/Y versus Ti/Y plot adapted from Pik et al.[5]. Various fields of spatially associated basalt suites are shown for comparison: northern Woranso-Mille basalts from west-central Afar[6], Pliocene to Quaternary mafic lavas from southern Afar and northern Main Ethiopian Rift[7,8,9], and Oligocene to Miocene mafic lavas and dikes from the northwestern Ethiopian plateau[10,5,11].

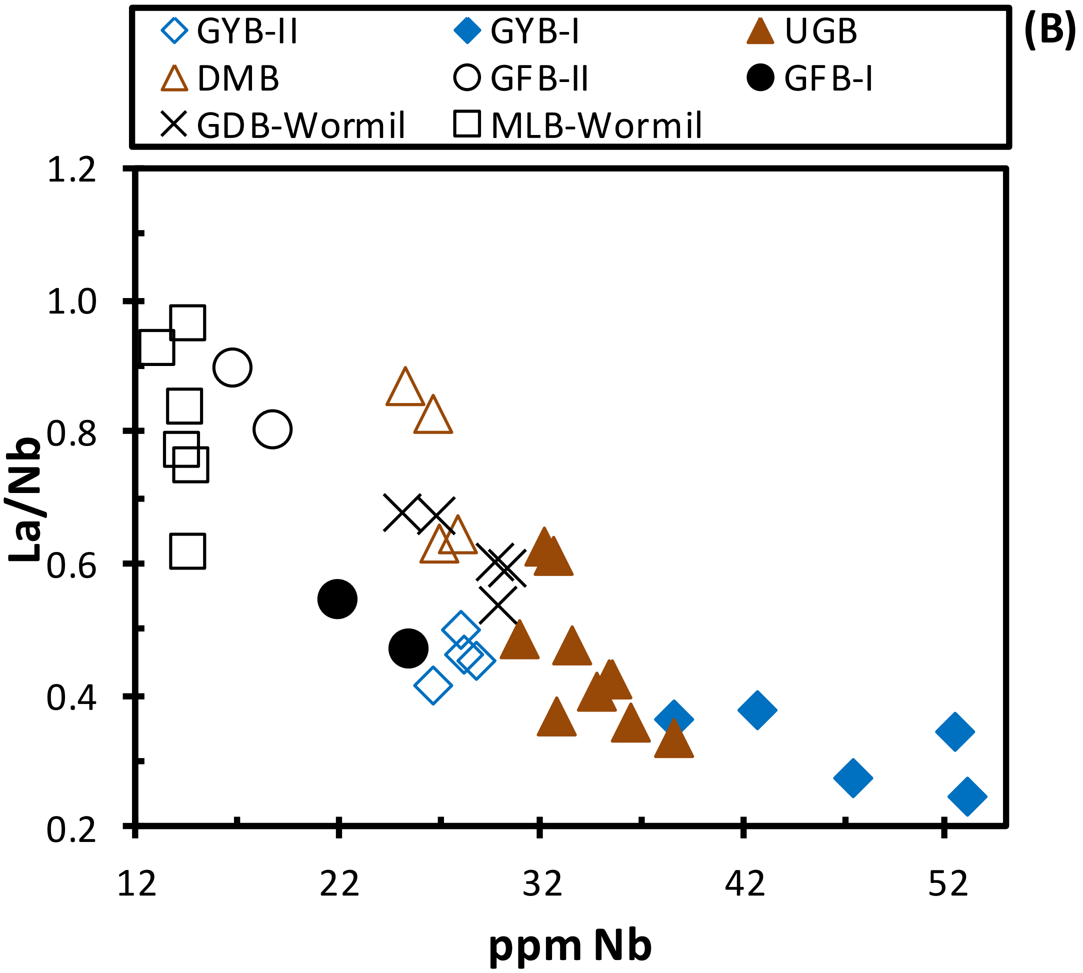

Supplementary Figure 2. La/Nb versus Nb plot. GDB-Wormil= Guda Basalt from Woranso-Mille, west-central AfarMLB-Wormil= Mille Basalt from Woranso-Mille, west central Afar[6].

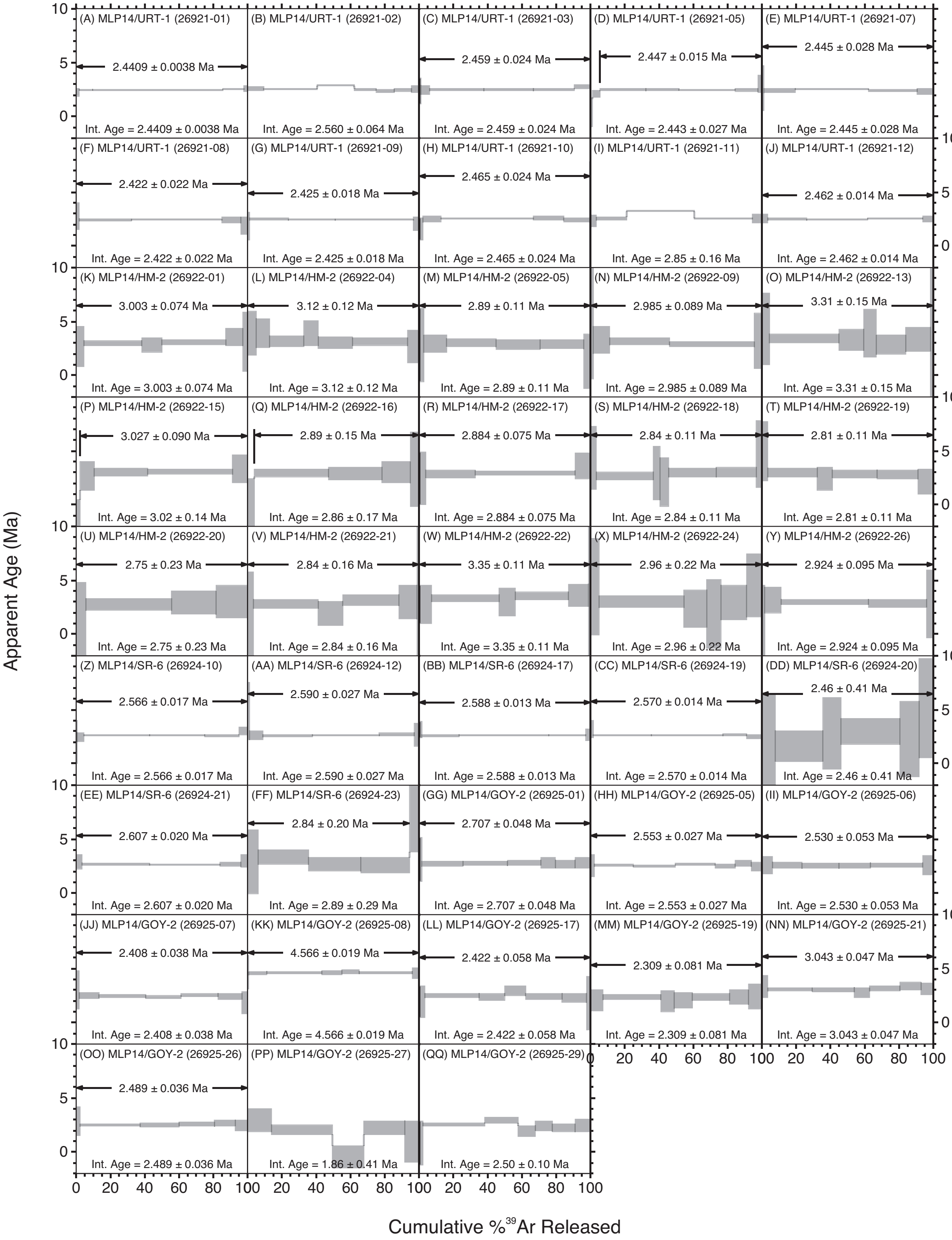

Supplementary Figure 3. Apparent age spectra from the completed SCIH experiments. Errors are shown at ±1σ, and do not include uncertainty in *J*, the neutron fluence parameter.

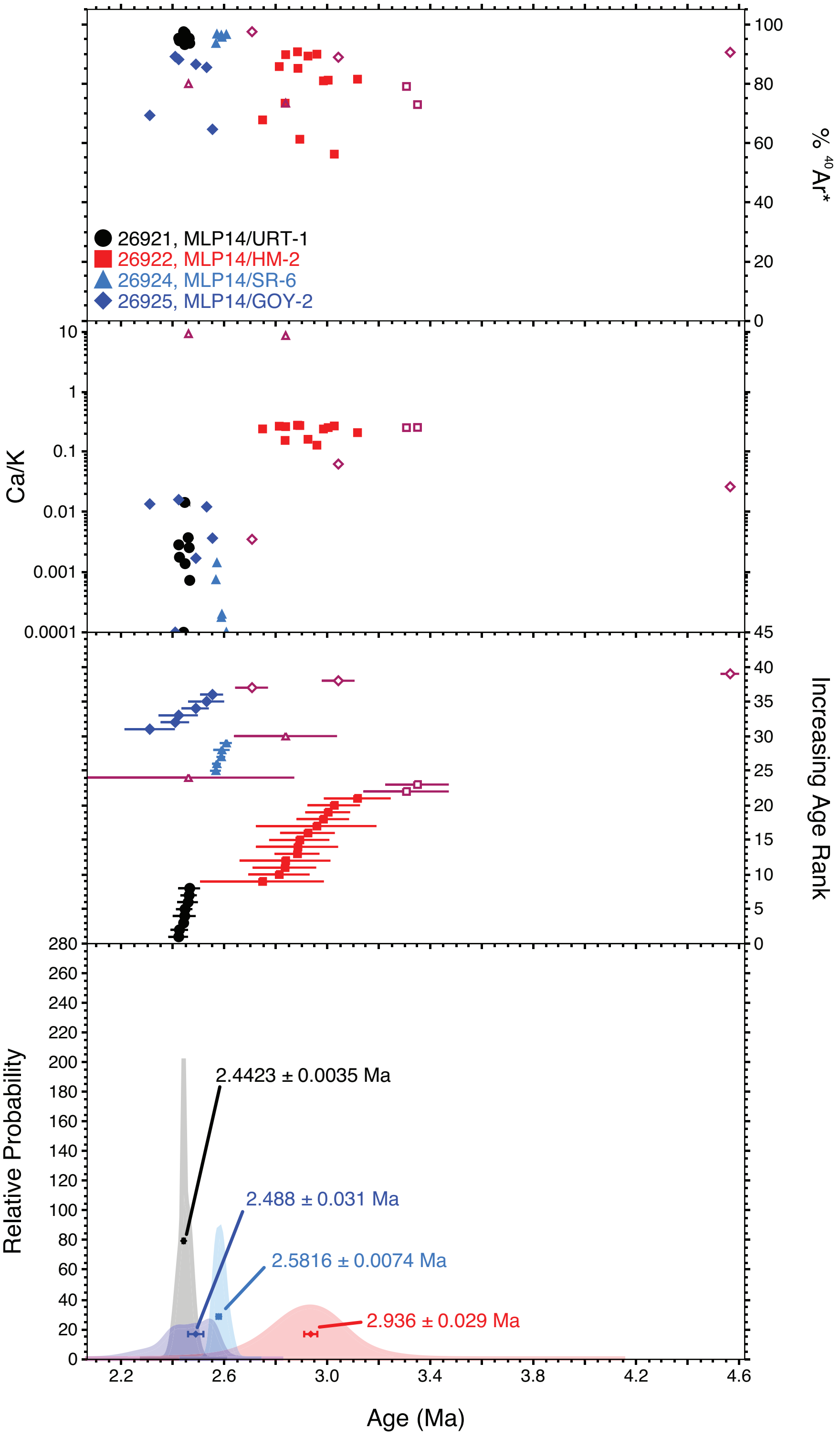

Supplementary Figure 4. Age-probability density spectra of the SCIH plateau ages. Open symbols denote omitted results. '% ^{40}Ar*' is the radiogenic percentage of total ^{40}Ar in the plateau steps, and 'Ca/K' is the atomic ratio calculated from the ^{37}Ar and ^{39}Ar isotopic composition of the plateau sequence. The weighted-mean age of the included plateau analyses is shown for each sample's primary mode in the lower panel (± 1σ, including error in *J*).

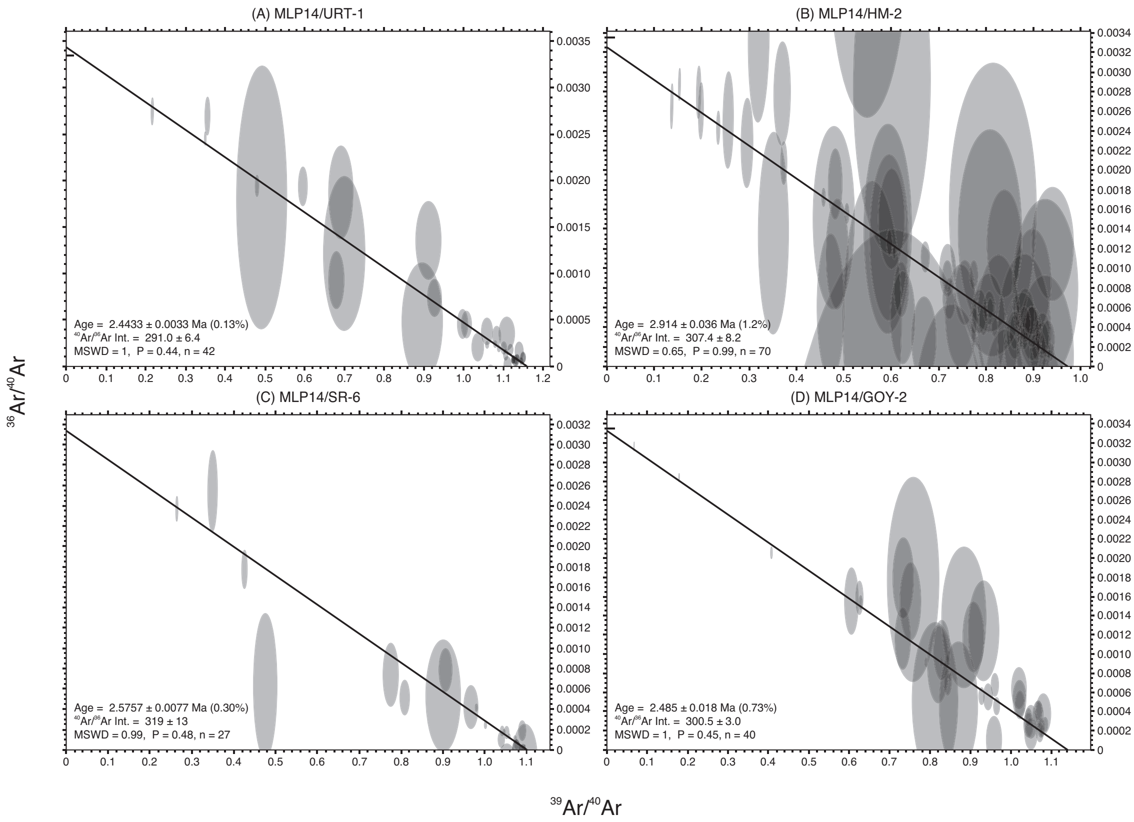

Supplementary Figure 5.‘Inverse-isochron’ analysis of the combined plateau steps of all grains attributable to each sample’s primary mode. The isochron result is considered here the best representative age for these samples (± 1σ, including error in *J*).

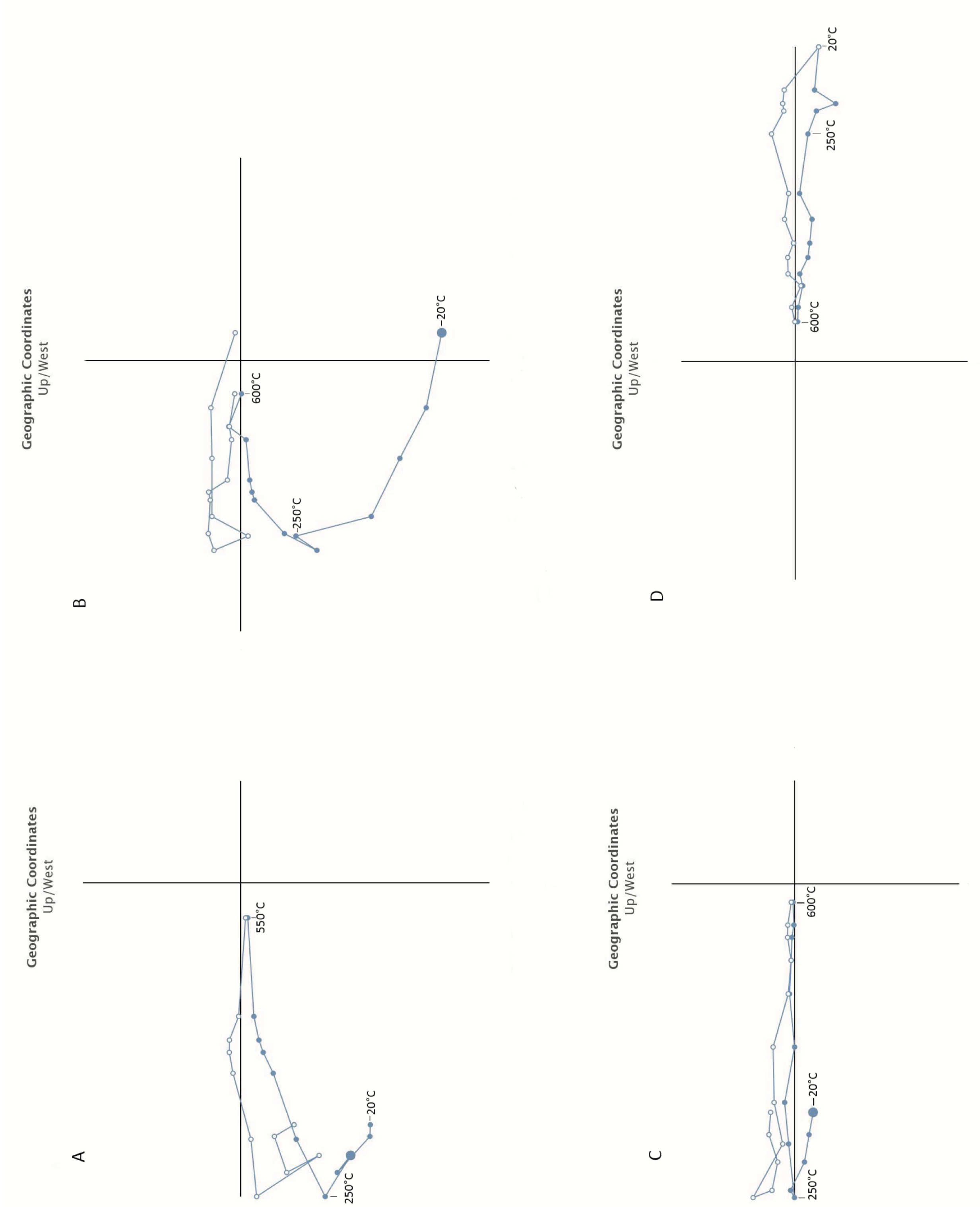

Supplementary Figure 6. Typical Zijderveld diagram[12] examples of two quality 1 samples, SL10 and SC10 with a normal and reversed ChRM direction (a, b). Zijderveld diagrams of SC04 is of quality 2 and has a reversed ChRM direction and GFB15-1 show a reversed ChRM direction of quality 1 (c, d). See main text for details. Closed/open circles denote projection on a horizontal/vertical plane.

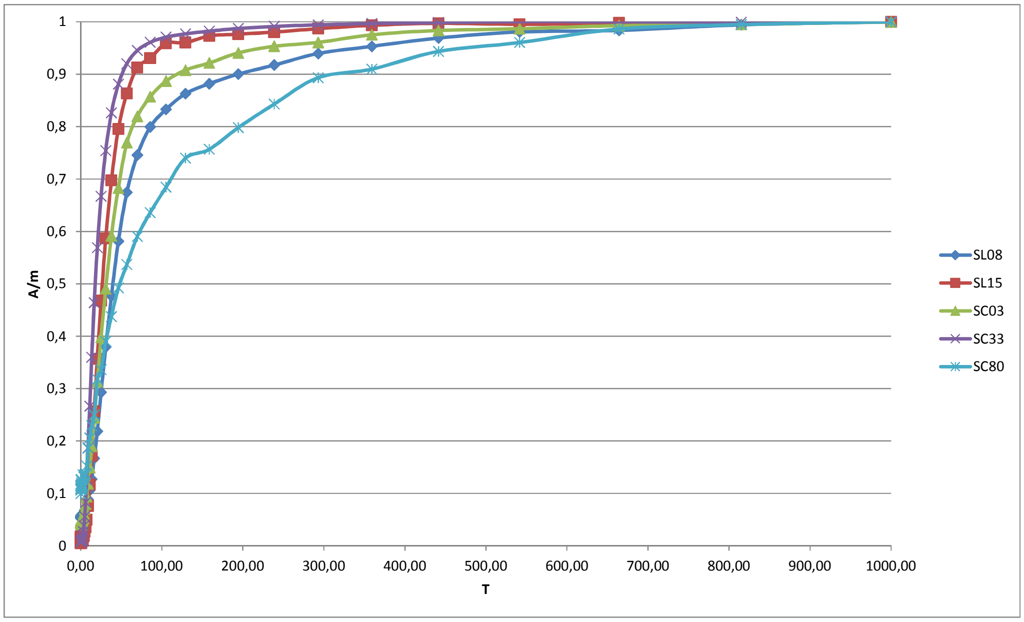

Supplementary Figure 7. Isothermal remanent magnetization (IRM) acquisition curve up to 1T for specimens SL08, SL15, SC03, SC33 and SC80

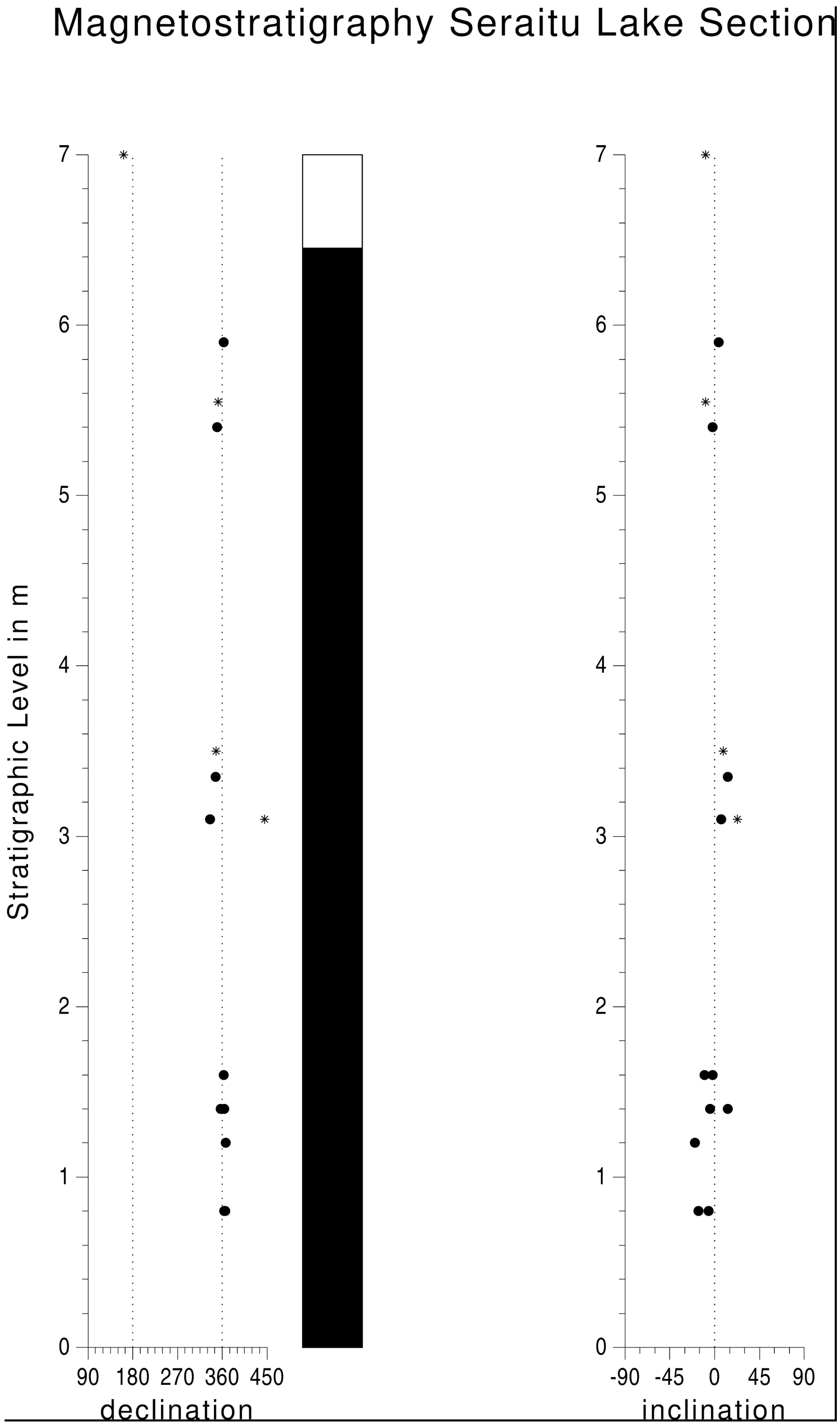

Supplementary Figure 8. Magnetostratigraphic results of the SL section. ChRM directions plotted against stratigraphic height in meters. Quality 1 directions are plotted in black dots, quality 2 samples are plotted in stars. See methods section in main text for further explanations.

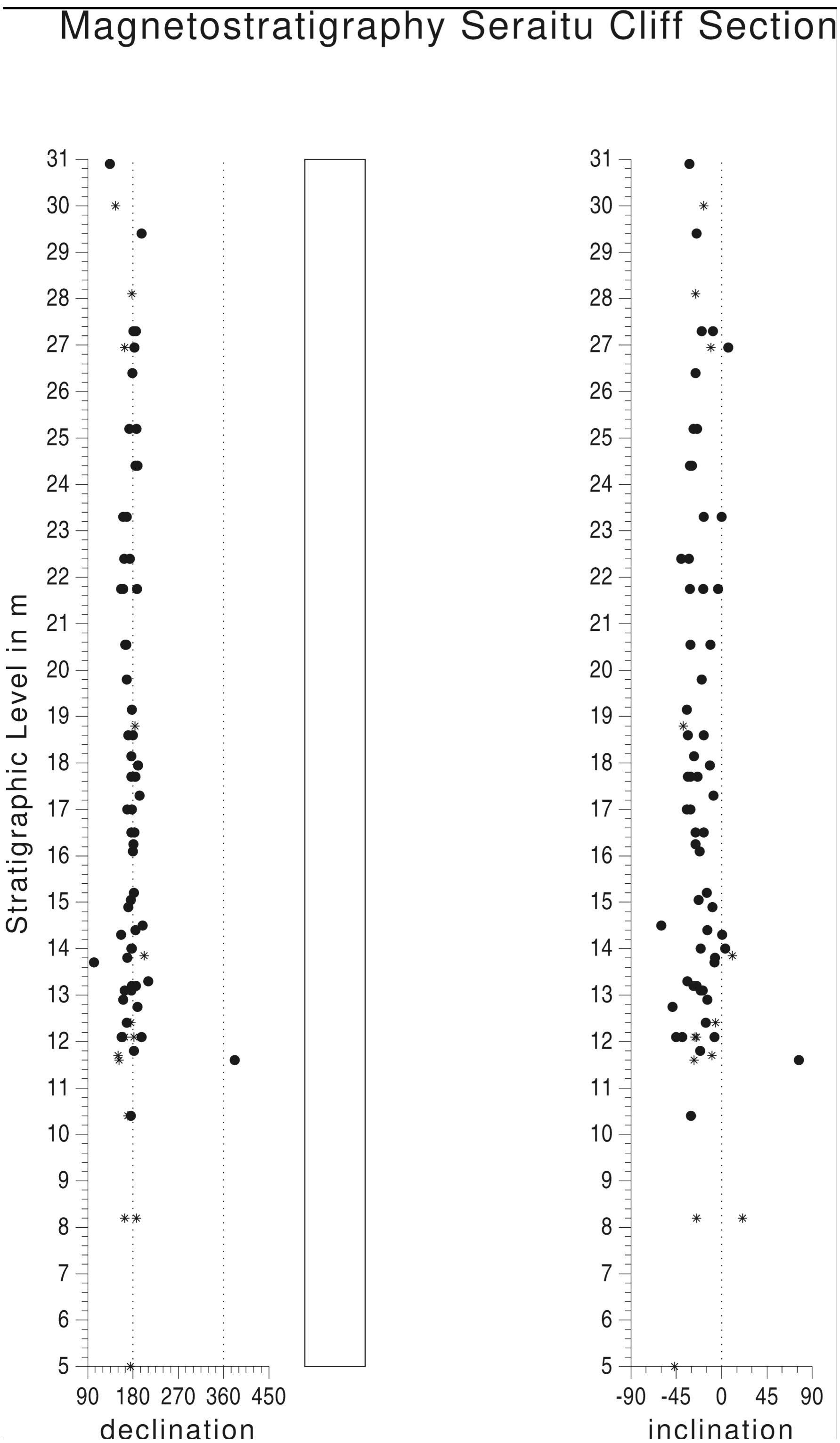

Supplementary Figure 9. Magnetostratigraphic results of the SC section. ChRM directions plotted against stratigraphic height in meters. Quality 1 directions are plotted in black dots, quality 2 samples are plotted in stars. See methods section in main text for further explanations.

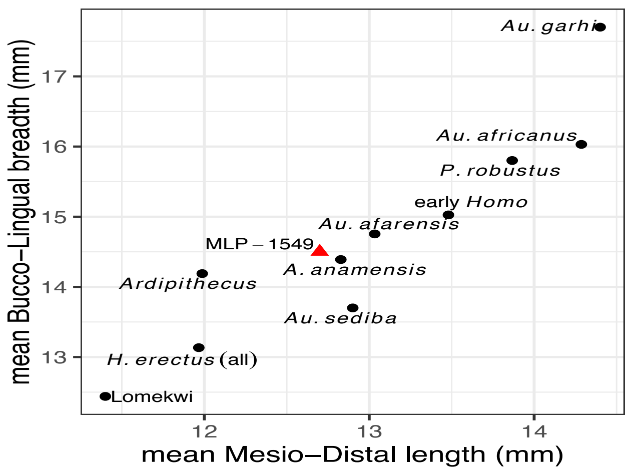

Supplementary Figure 10. MLP-1549 bucco-lingual breadth and mesio-distal length in mm compared to mean values for many hominin species.

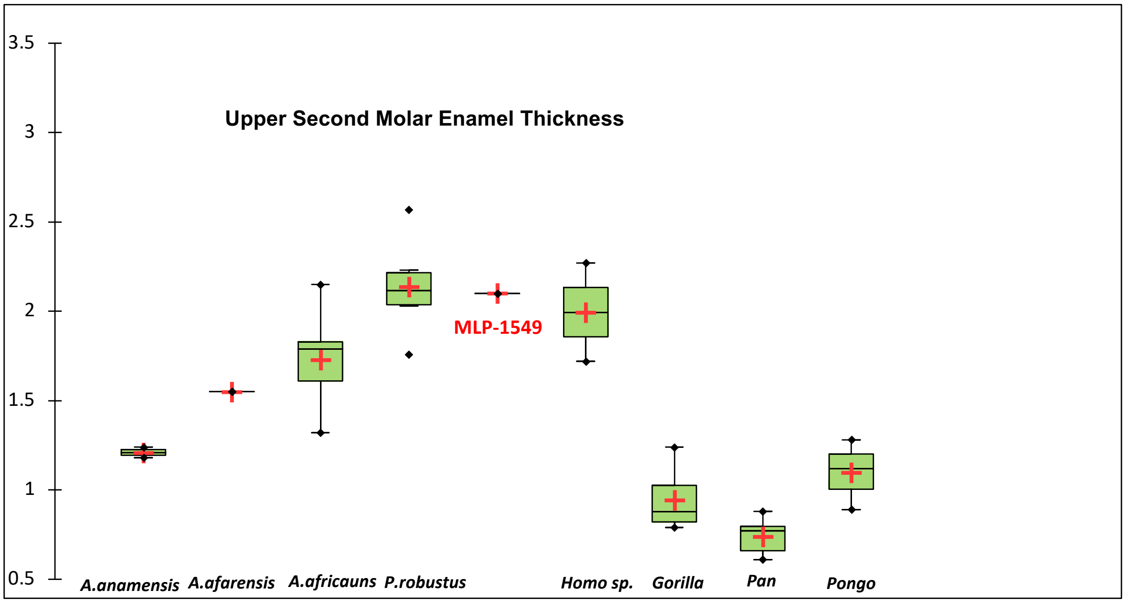

Supplementary Figure 11. MLP-1549 enamel thickness compared to values for other hominins. Source data are provided as a Source Data file.

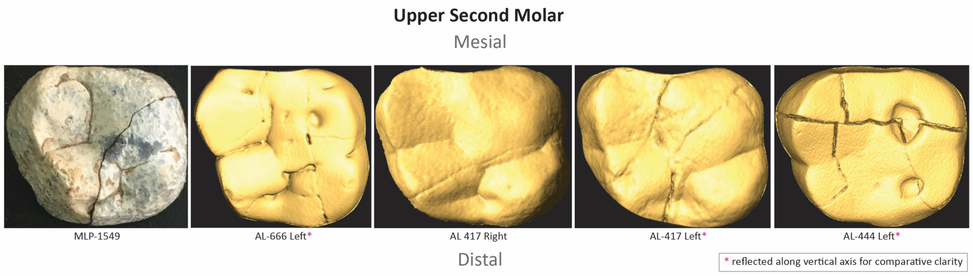

Supplementary Figure 12. Occlusal view of MLP-1549 compared that that of A.L. 666, A.L.417, A.L-444.

Supplementary References

1. Behrensmeyer, A.K., Potts, R., and Deino, A., The Oltulelei Formation of the southern Kenyan Rift Valley: a chronicle of rapid landscape transformation over the last 500 kyr. The Geological Society of America Bulletin. (in press)
2. Gradstein, F.M., Ogg, J.G., Schmitz, M.D., Ogg, G.M. (Eds.) The Geologic Time Scale, in: The Geologic Time Scale. *Elsevier*, p. iii. (2012) https://doi.org/10.1016/B978-0-444-59425-9.01001-5
3. Lisiecki, L.E., Raymo, M.E. A Pliocene-Pleistocene stack of 57 globally distributed benthic δ 18O records. *Paleoceanography*. **20**, 1-17 (2005) https://doi.org/10.1029/2004PA001071
4. Deino, A.L., King, J.W., Glen, J.M., Edgar, R.K., Hill, A. Precessional forcing of lacustrine sedimentation in the late Cenozoic Chemeron Basin, Central Kenya Rift, and calibration of the Gauss/Matuyama boundary. *Earth Planet. Sci. Lett*. **247**, 41–60 (2006) https://doi.org/10.1016/j.epsl.2006.04.009
5. Pik, R., Deniel, C., Coulon, C., Yirgu, G., Hoffmann, C., Ayalew, D. The northwestern Ethiopian Plateau flood basalts: classification and spatial distribution of magma types. *Journal of Volcanology and Geothermal Research* **81**, 91-111. (1998)
6. Alene, M., Hart, W.K., Saylor, B.Z., Deino, A., Mertzman, S., Haile-Selassie, Y., Gibert, L.B. Geochemistry of Woranso-Mille Pliocene Basalts from West-Central Afar, Ethiopia: Implications for Mantle Source Characteristics and Rift Evolution. *Lithos* **282-283**, 187-200. (2017) DOI: 10.1016/j.lithos.2017.03.005
7. Ayalew, D., Jung, S., Romer, R.L., Kersten, F., Pfander, J.A., Garbe-Schonberg, D. Petrogenesis and origin of modern Ethiopian rift basalts: constraints from isotope and trace element geochemistry. *Lithos* **258-259**, 1-14. (2016)
8. Rooney, T., Furman, T., Bastow, I.D., Ayalew, D., Gezahegn, Y. Lithospheric modification during crustal extension in the Main Ethiopian Rift. *Journal of Geophysical Research, B, Solid Earth and Planets* **112** (2007) doi:10.1029/2006JB004916.
9. Rooney, T.O., Bastow, I.D., Keir, D., Mazzarini, F., Movsesian, E., Grosfils, E.B., Zimbelman, J.R., Ramsey, M.WS., Ayalew, D., Yirgu, G. The protracted development of focused magmatic intrusion during continental rifting. *Tectonics* **33**, 875-897. (2014)

10. Kieffer, B., Arndt, N., LaPierre, H., Bastien, F., Bosch, D., Pecher, A., Yirgu, G., Ayalew, D., Weis, D., Jerram, D., Keller, F., Meugniot, C. Flood and shield basalts from Ethiopia: magmas from the African Superswell. *Journal of Petrology* **45**, 793-834. (2004)

11. Rooney, T.O., Mohr, P., Dosso, L., Hall, C. Geochemical evidence of mantle reservoir evolution during progressive rifting along the western Afar margin. *Geochimica et Cosmochimica Acta* **102**, 65-88 (2013)

12. Zijderveld, J.D.A. Ac Demagnetization of Rocks: Analysis of Results. *Methods in palaeomagnetism*. 254-286 (1967)
