# Supplementary material for: Fossils from Mille-Logya, Afar, Ethiopia, elucidate the link between Pliocene environmental changes and Homo origins
Source: Nat Commun. 2020 May 19;11:2480. doi: 10.1038/s41467-020-16060-8 (PMC7237685; doi:10.1038/s41467-020-16060-8)
Supplement: Supplementary file 3 — Reporting Summary [file 41467_2020_16060_MOESM3_ESM.pdf]

nature research

Corresponding author(s): Zeresenay Alemseged

Last updated by author(s): 2020/04/06

Reporting Summary

Nature Research wishes to improve the reproducibility of the work that we publish. This form provides structure for consistency and transparency in reporting. For further information on Nature Research policies, see Authors & Referees and the Editorial Policy Checklist .

Statistics

For all statistical analyses, confirm that the following items are present in the figure legend, table legend, main text, or Methods section.

n/a	Confirmed	
☐	☒	The exact sample size (*n*) for each experimental group/condition, given as a discrete number and unit of measurement
☒	☐	A statement on whether measurements were taken from distinct samples or whether the same sample was measured repeatedly
☒	☐	The statistical test(s) used AND whether they are one- or two-sided<br>*Only common tests should be described solely by name; describe more complex techniques in the Methods section.*
☒	☐	A description of all covariates tested
☒	☐	A description of any assumptions or corrections, such as tests of normality and adjustment for multiple comparisons
☒	☐	A full description of the statistical parameters including central tendency (e.g. means) or other basic estimates (e.g. regression coefficient) AND variation (e.g. standard deviation) or associated estimates of uncertainty (e.g. confidence intervals)
☒	☐	For null hypothesis testing, the test statistic (e.g. *F*, *t*, *r*) with confidence intervals, effect sizes, degrees of freedom and *P* value noted<br>*Give P values as exact values whenever suitable.*
☒	☐	For Bayesian analysis, information on the choice of priors and Markov chain Monte Carlo settings
☒	☐	For hierarchical and complex designs, identification of the appropriate level for tests and full reporting of outcomes
☒	☐	Estimates of effect sizes (e.g. Cohen's *d*, Pearson's *r*), indicating how they were calculated

Our web collection on statistics for biologists contains articles on many of the points above.

Software and code

Policy information about availability of computer code

Data collection	Field data collection utilized Garafa GIS Pro software version 4 and Paleo Core data management software version 0.2.
Data analysis	XLSTAT, paleomag software at paleomagnetism.org, geocrhon methods as cited in supplementary information; Map in this paper was generated with the standard distribution of QGIS version 3.10.2.

For manuscripts utilizing custom algorithms or software that are central to the research but not yet described in published literature, software must be made available to editors/reviewers. We strongly encourage code deposition in a community repository (e.g. GitHub). See the Nature Research guidelines for submitting code & software for further information.

Data

Policy information about availability of data

All manuscripts must include a data availability statement. This statement should provide the following information, where applicable:

- Accession codes, unique identifiers, or web links for publicly available datasets
- A list of figures that have associated raw data
- A description of any restrictions on data availability

Data are available in supplementary information. The source data underlying Fig. 4 and Supplementary Fig. 11 are provided as a Source Data file

Field-specific reporting

Please select the one below that is the best fit for your research. If you are not sure, read the appropriate sections before making your selection.

☐ Life sciences ☐ Behavioural & social sciences ☒ Ecological, evolutionary & environmental sciences

Ecological, evolutionary & environmental sciences study design

All studies must disclose on these points even when the disclosure is negative.

Study description	The study primarily involves field fossil and geological sample collection for comparative and dating purposes
Research sample	Research samples are typically characterized by vertebrate fossil remains and geological samples
Sampling strategy	Our research samples are typically characterized by vertebrate fossil remains and geological samples both collected following widely used traditional strategies.
Data collection	Field data collection utilized Garafa GIS Pro software version 4 and Paleo Core data management software version 0.2.
Timing and spatial scale	Data come from the Mille Logya area of the Afar region in Ethiopia and date back to between c.a. 2.4 to 2.9 Ma
Data exclusions	Radiometric isotope data were excluded from analysis as described in methods section.
Reproducibility	Replication of geochemical, geochronological and magnetostratigraphic data are described in methods section and supplementary information.
Randomization	No randomization was required.
Blinding	Investigators were not blinded during experiments.

Did the study involve field work? ☒ Yes ☐ No

Field work, collection and transport

Field conditions	Fieldwork pertains to fossil collection and traditional geological survey and sampling
Location	Mille-Logya area, Afar Regional State, Ethiopia
Access and import/export	Samples pertain to volcanic ashes for radiometric dating and tephra analysis and they were exported from Ethiopia to the US with permission from the Ethiopian Authorities
Disturbance	There was no disturbance

Reporting for specific materials, systems and methods

We require information from authors about some types of materials, experimental systems and methods used in many studies. Here, indicate whether each material, system or method listed is relevant to your study. If you are not sure if a list item applies to your research, read the appropriate section before selecting a response.

Materials & experimental systems

n/a	Involved in the study	
☒	☐	Antibodies
☒	☐	Eukaryotic cell lines
☐	☒	Palaeontology
☒	☐	Animals and other organisms
☒	☐	Human research participants
☒	☐	Clinical data

Methods

n/a	Involved in the study	
☒	☐	ChIP-seq
☒	☐	Flow cytometry
☒	☐	MRI-based neuroimaging

Palaeontology

Specimen provenance	We collect only fossil samples and permit is issued by the Authority for Research and Conservation of Cultural Heritage of Ethiopia
Specimen deposition	National Museums of Ethiopia with permission from the museum
Dating methods	Ar/Ar

☒ Tick this box to confirm that the raw and calibrated dates are available in the paper or in Supplementary Information.
